# Loss of polarity alters proliferation and differentiation in low-grade endometrial cancers by disrupting Notch signaling

**DOI:** 10.1371/journal.pone.0189081

**Published:** 2017-12-05

**Authors:** Erin Williams, Alejandro Villar-Prados, Jessica Bowser, Russell Broaddus, Andrew B. Gladden

**Affiliations:** 1 Department of Genetics, University of Texas, MD Anderson Cancer Center, Houston, TX, United States of America; 2 Program of Genes and Development, Graduate School of Biomedical Sciences, University of Texas Health Sciences Center, Houston, TX, United States of America; 3 Department of Translational Molecular Pathology, University of Texas, MD Anderson Cancer Center, Houston, TX, United States of America; 4 Department of Pathology, University of Texas, MD Anderson Cancer Center, Houston, TX, United States of America; NCMLS, Radboud University Nijmegen Medical Center, NETHERLANDS

## Abstract

Cell adhesion and apicobasal polarity together maintain epithelial tissue organization and homeostasis. Loss of adhesion has been described as a prerequisite for the epithelial to mesenchymal transition. However, what role misregulation of apicobasal polarity promotes tumor initiation and/or early progression remains unclear. We find that human low-grade endometrial cancers are associated with disrupted localization of the apical polarity protein Par3 and Ezrin while, the adhesion molecule E-cadherin remains unchanged, accompanied by decreased Notch signaling, and altered Notch receptor localization. Depletion of Par3 or Ezrin, in a cell-based model, results in loss of epithelial architecture, differentiation, increased proliferation, migration and decreased Notch signaling. Re-expression of Par3 in endometrial cancer cell lines with disrupted Par3 protein levels blocks proliferation and reduces migration in a Notch dependent manner. These data uncover a function for apicobasal polarity independent of cell adhesion in regulating Notch-mediated differentiation signals in endometrial epithelial cells.

## Introduction

Loss of epithelial architecture is a hallmark of cancer that is regularly used to diagnose the presence of disease. Epithelial architecture is established through cell:cell and cell:matrix contacts that organize and form specialized tissues. Initiation of cell:cell contacts at adherens junctions (AJ) serves as a molecular landmark to promote establishment of apicobasal polarity and organization of distinct plasma membrane domains on the apical, basal and lateral surfaces of the cell [1,2]. Apicobasal polarity is conserved in all metazoans and is necessary for correct organization of tissues and proper organ function [3]. Disruption of apicobasal polarity is generally associated with many advanced cancers and is thought to be associated with loss of cell adhesion [4]. However, whether mis-regulation of apicobasal polarity promotes tumor initiation and/or early progression remains unclear. Protein complexes including the Par3/aPKC apical polarity complex and basal polarity proteins establish a gradient that promotes polarization of epithelial cells [3,5,6]. Recent work showed that depletion of Par3 in mouse mammary glandular epithelium cooperated with known oncogenic signaling pathways to promote mammary tumor progression [7]. We aimed to determine whether loss of apicobasal polarity occurs in other glandular epithelial cancers, specifically endometrial tissue.

The epithelium of the endometrium, the primary cell type thought to give rise to cancer, is shed and re-established after each menstrual cycle [8,9]. This continual repopulation requires a constant reorganization of cell:cell junctions and apicobasal polarity that is essential for reproductive function of the endometrial epithelium [1,10–12]. In addition to proper organization of the endometrial epithelium, the menstrual cycle and reproductive abilities also require differentiation of the epithelial cells [8,9]. Deregulation of differentiation, at the cellular level of the epithelium and at the level of the tissue, is thought to lead to the development of endometrial cancer. Endometrial cancer is the fourth most common cancer in women in the U.S. and generally arises from neoplastic epithelial cells [13–16]. Endometrioid endometrial carcinoma (EEC) is the most common histotype, accounting for 80% of endometrial cancers [17,18]. EEC is commonly categorized by grade (G1, G2, and G3, or well-, moderately-, and poorly-differentiated, respectively), which is based on tumor differentiation and percent solid growth [19]. G1 EEC have well-formed glands and <5% solid tumor growth while G3 EEC have more than 50% solid growth and lack well-formed glands [19]. Tumor grade has prognostic significance in endometrial cancer [13]; G1 EECs rarely metastasize and generally have a good prognosis compared to G3 EECs. The PI3K/PTEN and Ras/Raf/MEK signaling pathways are often altered in endometrial cancer [20]. Additional molecular pathway(s) disrupted in endometrial tumorigenesis are less clear [21]. Understanding how epithelial differentiation and apicobasal polarity is regulated in the endometrium could lead to a better understanding of the mechanisms of endometrial tumor development and progression.

Notch signaling is a core pathway involved in the differentiation of many types of tissue in different organisms including the human endometrium [22–24]. Activation of Notch signaling begins with stimulation of membrane-bound receptors by transmembrane ligands which triggers a signaling cascade that leads to increased transcription of Notch downstream targets [25]. This signaling pathway is thought to be highly regulated in the endometrium, particularly during different phases of the menstrual cycle [22]. In cancerous tissues, Notch signaling displays tissue-specific, tumor suppressive or oncogenic properties [26–28]. In T-cell acute lymphoblastic leukemia, Notch displays oncogenic properties: activating mutations in Notch receptors drive downstream signaling that promotes proliferation [26]. However, Notch acts as a tumor suppressor in keratinocytes where Notch deficiency causes increased proliferation and decreased differentiation leading to basal cell carcinoma-like tumors [28,29]. While a variety of studies have examined how aberrant levels of Notch pathway activity can regulate tissue homeostasis [30–32], there is little known about the involvement of Notch receptor or ligand subcellular localization in tumorigenesis. Here, we examine the status of apicobasal polarity in low-grade EEC and demonstrate a link between apicobasal polarity, Notch receptor localization, and differentiation.

## Materials and methods

### Immunohistochemistry

Use of human tissues was approved by the IRB of the University of Texas MD Anderson Cancer Center (LAB1-718) (PI:Broaddus). Informed written consent was obtained through the MD Anderson Cancer Center “Front Door” tissue banking consent policy (LAB03-0320). All authors received training on working with and the ethics of human specimen research from MD Anderson Cancer Center. De-identified primary human endometrial tissue samples were obtained from the tissue biospecimen and pathology core facility at MD Anderson Cancer Center. Frozen endometrial tissue sections were immediately fixed in 4% paraformaldehyde/PBS for 20 minutes at room temperature. Samples were permeabilized using 0.5% Triton in PBS for 15 minutes at room temperature and then blocked for 30 minutes in blocking buffer containing 10% fetal bovine serum (FBS) and 1% bovine serum albumin (BSA) diluted in PBS-0.1% Tween 20 (PBST). Sections were subsequently incubated with the following primary antibodies: Par3 (1:500, rabbit, Millipore, 07–330), Ezrin (1:500, mouse, Invitrogen, 3C12), E-cadherin (1:1000, mouse, BD, 610182), Acetylated Tubulin (1:1000, mouse, Sigma, T6793), Pan Cytokeratin (1:500, rabbit, Abcam, PA5-21985), and Pericentrin (1:1000, rabbit, Abcam, ab4448) overnight at 4°C. Slides were then washed three times in PBST and incubated with secondary antibodies CY3 Rabbit (1:200, Jackson Immunoresearch), Alexa 488 Mouse (1:200, Jackson Immunoresearch), Phalloidin CY5 (1:250, Invitrogen), and 4’6’-diamidino-2-phenylindole (DAPI,1:1000). Slides were washed two times in PBST, one time in PBS and mounted with Vectashield (Vector Laboratories).

Staining of MDCK 3D cultures was adapted from previous descriptions [2,33], briefly, cultures were fixed in 3.7% Formalin in cytoskeletal buffer (10 mM 2-(N-morpholino)-ethanesulfonic acid sodium salt (MES) pH 6.1, 138 mM KCl, 3 mM MgCl_2_, 2 mM EGTA) for 20 minutes at room temperature. Washed in PBS and then permeabilized in 0.5% Triton/PBS for 20 minutes at room temperature. Cultures were then washed in PBS and subsequently washed with 100 mM Glycine/PBS for 15 minutes, 3 times. Cultures were blocked for 1 hour in PBST/0.2% Triton/1% BSA/10% FBS and then left overnight at room temperature with primary antibodies in blocking buffer. Following primary antibody incubation cultures were washed in PBST 3 times for 20 minutes and then incubated in blocking buffer with secondary antibodies for 1 hour at room temperature. All tissue samples and labeled cells were visualized using a Nikon A1 laser scanning confocal microscope.

### Cell culture and reagents

Madin-Darby Canine Kidney (MDCK) cell lines and endometrial cancer cell lines were cultured in Dulbecco’s Modified Eagle’s Medium (DMEM) with 10% fetal bovine serum (FBS). Generation of knockdown MDCK cell lines is described below. Knockdown MDCK cells were selected with puromycin (7 μg/mL). Re-expression cell lines were selected with hygromycin (30 μg/mL). The gamma-secretase inhibitor DAPT (Sigma) was placed on cells at a concentration of 1 μg/ml in new media and cells were provided fresh DAPT and media every 24 hours for 48 hours for BrdU and expression experiments and up to 72 hours for migration experiments.

### 3D culture assay

MDCK 3D cyst formation was modified from previously described work [34]. In short, an 8-well chamber slide was pre-coated with a collagen mixture (24 μM Glutamine, 2.35 mg/mL NaHCO_3_, 1x MEM, 20 mM HEPES, 2 mg/mL Collagen I). MDCK cells were suspended in the same collagen mixture at a concentration of 3x10^4^ cells per ml of collagen mixture. DMEM with 10% FBS was added 45 minutes after placing the chamber slide in the 37°C incubator. The DMEM media was changed every 2–3 days without disturbing the matrix/cell layer.

### Generation of knockdown MDCK cell lines

Virus was produced by transfecting HEK293T cells at 80–90% confluency with the pLK0.1 lentiviral expression vector obtained from Open biosystems (shRNA- Par3 clone ID: TRCN0000118134 or shRNA-Ezrin clone ID: TRCN0000062462) and packaging vectors (VSVG and ΔVPR). MDCK cells were infected with the virus and polybrene (1 μg/mL) in DMEM with 10% FBS. The following day, new media was added with puromycin (7 μg/mL) for selection. Knockdown was verified by Western blots.

### Generation of overexpression endometrial cancer cell lines

Twenty-four hours prior to transfection, cells were plated at 2x10^5^ on each well of a 6-well dish. Par3-myc was transfected in when the cells reached 50–70% confluency using Lipofectamine LTX (Invitrogen) and Plus Reagent (Invitrogen) in DMEM without FBS. The Par3-myc construct was obtained from the Macara lab and has been previously described [35]. Twenty-four hours post-transfection, fresh DMEM with 10% FBS was added to cells. Cells were fixed 48 hours after original transfection.

### Quantitative RT-PCR

For Human Endometrial Tissue Samples: The qRT-PCR was performed as indicated by Schlumbrecht et al. [36]. For MDCK cells: RNA was extracted using Trizol and chloroform. RNA was purified with 100% isopropanol and 75% ethanol. RNA pellets were dissolved in 30–50 μL of DEPC water at 55°C. RNA purity was confirmed by nanodrop to have an A260/A280 ratio greater than 1.8. cDNA was synthesized using a SuperScript First-Strand Synthesis kit. The cDNA was analyzed using SYBR green quantification with the 7900HT Sequence Detection System (Applied Systems). Primers used are found in the table, (S1 Table). Samples were assayed in triplicate. Individual data points are available online in S1 Dataset. Data were normalized to HPRT. Samples below the limit of quantification were not included. The fold change of the ΔCT compared to HPRT was utilized for analysis (2^-ΔCT^).

### Cell extract preparation and Western blotting

For Western blots, cells were scraped off culture dishes and suspended in Triton lysis buffer (1% Triton X-100, 50 mM Tris-HCl pH 7.4, 140 mM NaCl, 1 mM EDTA, 1 mM EGTA, 1 mM PMSF, 1 mM Na3VO4, 1 mM sodium fluoride, 1 mM β-glycerophosphate, 10 μg/mL aprotinin, 10 μg/mL leupeptin) followed by sonication. Cell lysates went through centrifugation and the debris was removed. Bradford assays were used to quantify the amount of proteins. Cell lysates (30 μg total protein) were loaded onto SDS-PAGE gels and transferred to a PVDF membrane. Membrane blots were blocked for at least 1 hour in 5% milk in TBST. Primary antibody was added overnight at 4°C. Primary antibodies include: Par3 (1:750, rabbit, Millipore, 07–330), Ezrin (1:1000, mouse, Invitrogen, 3C12), Notch1 (1:500, rabbit, Cell signaling, D1E11), Notch2 (1:750, rabbit, Cell signaling, D76A6), and β-tubulin (1:1000, Sigma/Santa Cruz, T7816/sc-9104). Secondary antibodies were put on the next day for at least 30 minutes. Protein expression was depicted using enhanced chemiluminescence on a LiCor machine.

### Image processing, analysis, and densitometry

All tissue samples and cells were visualized using a Nikon A1 laser scanning confocal microscope. Images were processed using the Nikon-Elements software (Nikon). Quantitative analysis of endometrial tumor samples was performed blind by an independent investigator. Densitometry was performed by Image Studios Version 3.1 using the data from the LiCor machine.

## Results

### Loss of polarity, but not E-cadherin localization, in low-grade endometrial cancer

**Fig 1:**
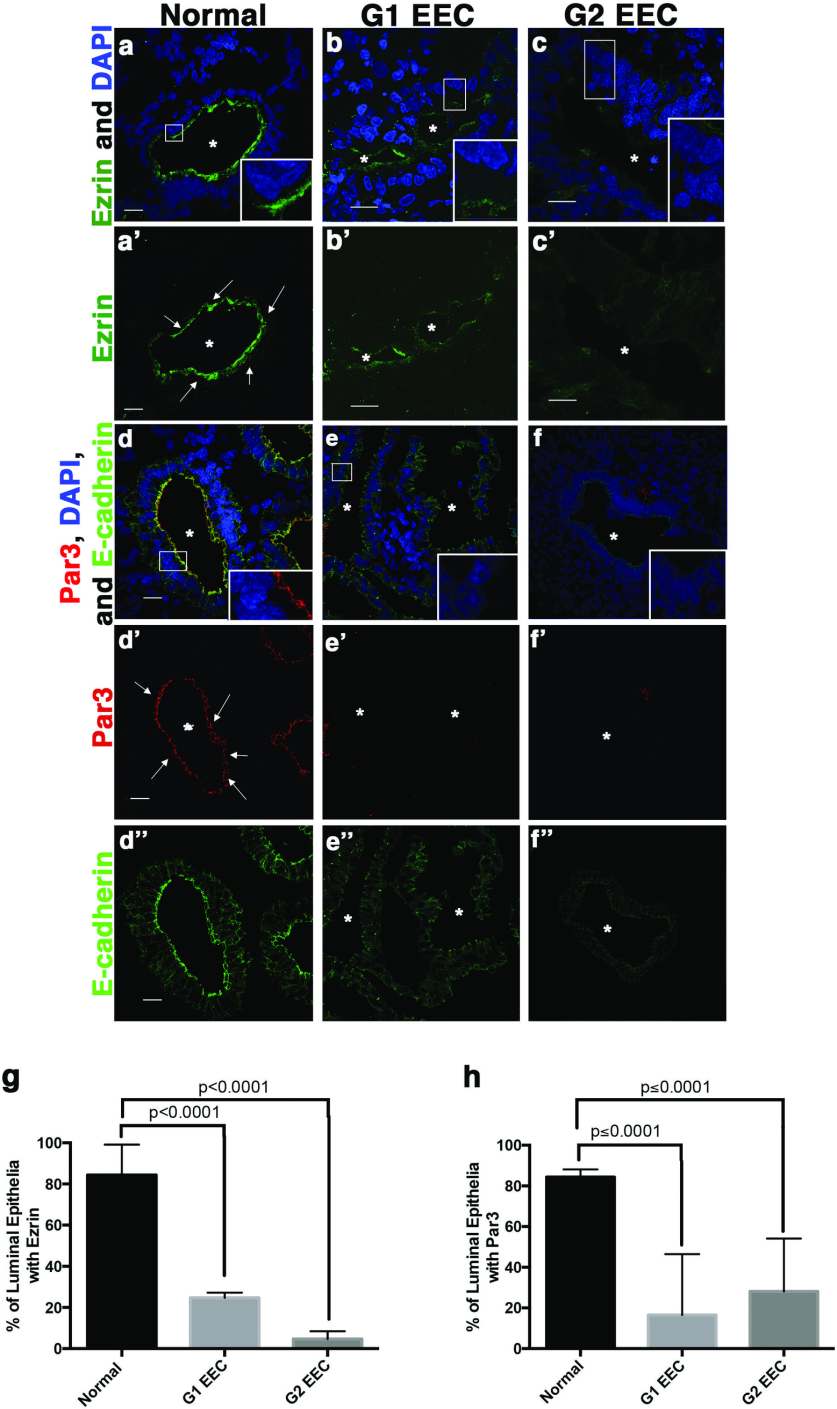
Loss of apicobasal polarity occurs in low-grade endometrial cancer. Human endometrial tissue (**a, a’, d, d’, d”**), normal; (**b, b’, e, e’, e”**) grade 1 endometrioid endometrial carcinoma, G1 EEC; and (**c, c’, f, f’, f”**) grade 2 endometrioid endometrial carcinoma, G2 EEC stained with antibodies for the apical proteins (**a-c**) Ezrin or (**d-f**) Par3, E-cadherin and DAPI. Scale bar, 20 μm. Asterisks indicate glandular lumen and arrows show apical localizing protein (**a’**) Ezrin or (**d’**) Par3. Scale bar, 20 μm. (**g and h**) Quantification of (**a-c**) Ezrin or (**d-f**) Par3 apical localization in 10 lumens of each sample (n = 3 normal, n = 2 G1 EEC, n = 2 G2 EEC) showing loss of apical protein localization in low-grade EEC. Error bars represent SEM.

Normal human endometrium consists of a single layer of polarized glandular epithelium resting on the adjacent stroma [37]. To determine the status of apicobasal polarity in EEC, we first examined the localization of the apical protein, Ezrin or the apical polarity protein, Par3 in normal endometrium in comparison to the localization of the adherens junction protein E-cadherin. Both Par3 and Ezrin localized to the apical side of the polarized glandular epithelium of normal endometrium (Fig 1A, 1A’, 1D and 1D’). We next examined Ezrin and Par3 localization in G1 EEC (Fig 1B, 1B’, 1E and 1E’) and G2 EEC (Fig 1C, 1C’, 1F and 1F’) endometrial cancer samples. We observed decreased apical localization of both Par3 and Ezrin in glandular structures of the tumors compared to little change in E-cadherin localization (Fig 1D”, 1E” and 1F”). Quantification of the percentage of glandular epithelial cells with apical localization of either Ezrin (Fig 1G) or Par3 (Fig 1H) shows nearly a four-fold decrease in EECs compared to normal endometrium. Additionally, the presence of the apically localized differentiation marker acetylated tubulin which marks cilia [38–40], was decreased in G1 and G2 EEC (S1 Fig). These data indicate that apicobasal polarity is disrupted in low-grade endometrial tumors.

**Fig 2:**
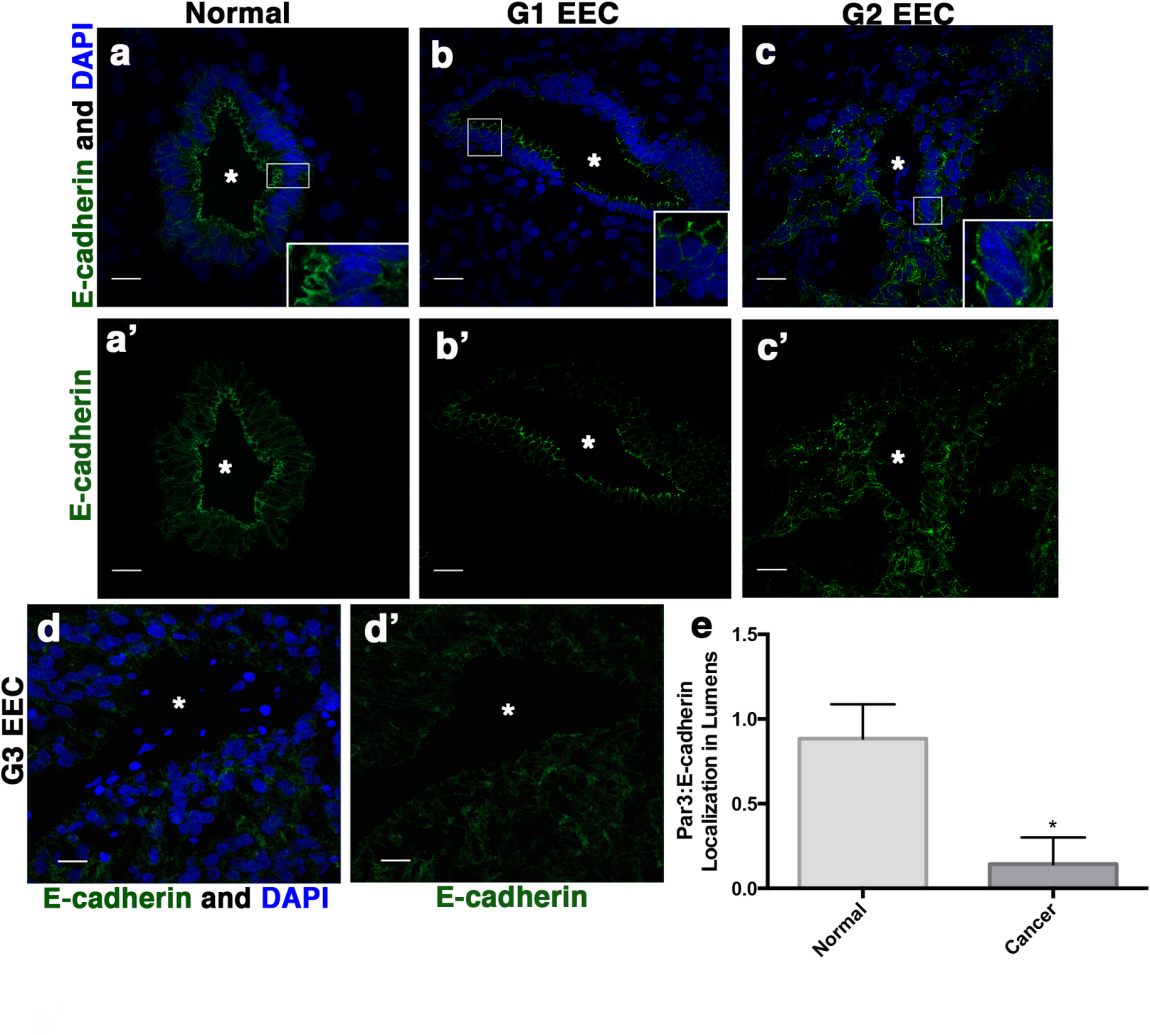
E-cadherin localization in endometrial cancer with disrupted polarity. (**a, a’**) Normal endometrium, (**b, b’**) G1 EEC and (**c, c’**) G2 EEC stained with an antibody against E-cadherin and DAPI. (**a’-c’**) Images of (**a-c**) E-cadherin staining showing localization to the apical junctions and lateral border in normal endometrium, G1 EEC, and G2 EEC. Asterisks indicate glandular lumen. (**d**) G3 EEC stained with antibodies against E-cadherin (green) with DAPI or (**d’**) with E-cadherin staining only showing loss of localization to the apical junctions and lateral borders. (**e**) Ratio of the apical localization of Par3 to the basolateral localization of E-cadherin. Localization of Par3 or E-cadherin was determined from 10 lumens of each sample (n = 3 normal, n = 4 EEC). Error bars represent SEM. * <0.05. Scale bar, 20 μm.

We previously demonstrated that establishment of apicobasal polarity in developing epithelial tissue requires AJ formation [1]. Loss of polarity is closely associated with advanced or metastatic tumors and epithelial-to-mesenchymal transition (EMT), a transcriptional program that down-regulates the AJ protein, E-cadherin [4]. To investigate E-cadherin protein expression and localization in G1 and G2 EECs lacking apicobasal polarity, we stained normal endometrium and EECs for E-cadherin. Interestingly, although apicobasal polarity was disrupted (Fig 1), E-cadherin remained present and localized to the basolateral membranes of glandular epithelial cells in G1 and G2 EEC (Fig 2A–2C, 2A’–2C’ and 2E). Subsequently in more advanced G3 EEC, we see loss of E-cadherin expression (Fig 2D and 2D’) indicating loss of E-cadherin is a late event in EEC. Overall, these data demonstrate that loss of apicobasal polarity, but not the AJ marker E-cadherin, corresponds with decreased cellular differentiation in low-grade endometrial tumors.

### Disruption of polarity in a 3D cell model phenocopies changes in cellular differentiation observed in low-grade endometrial tumors

**Fig 3:**
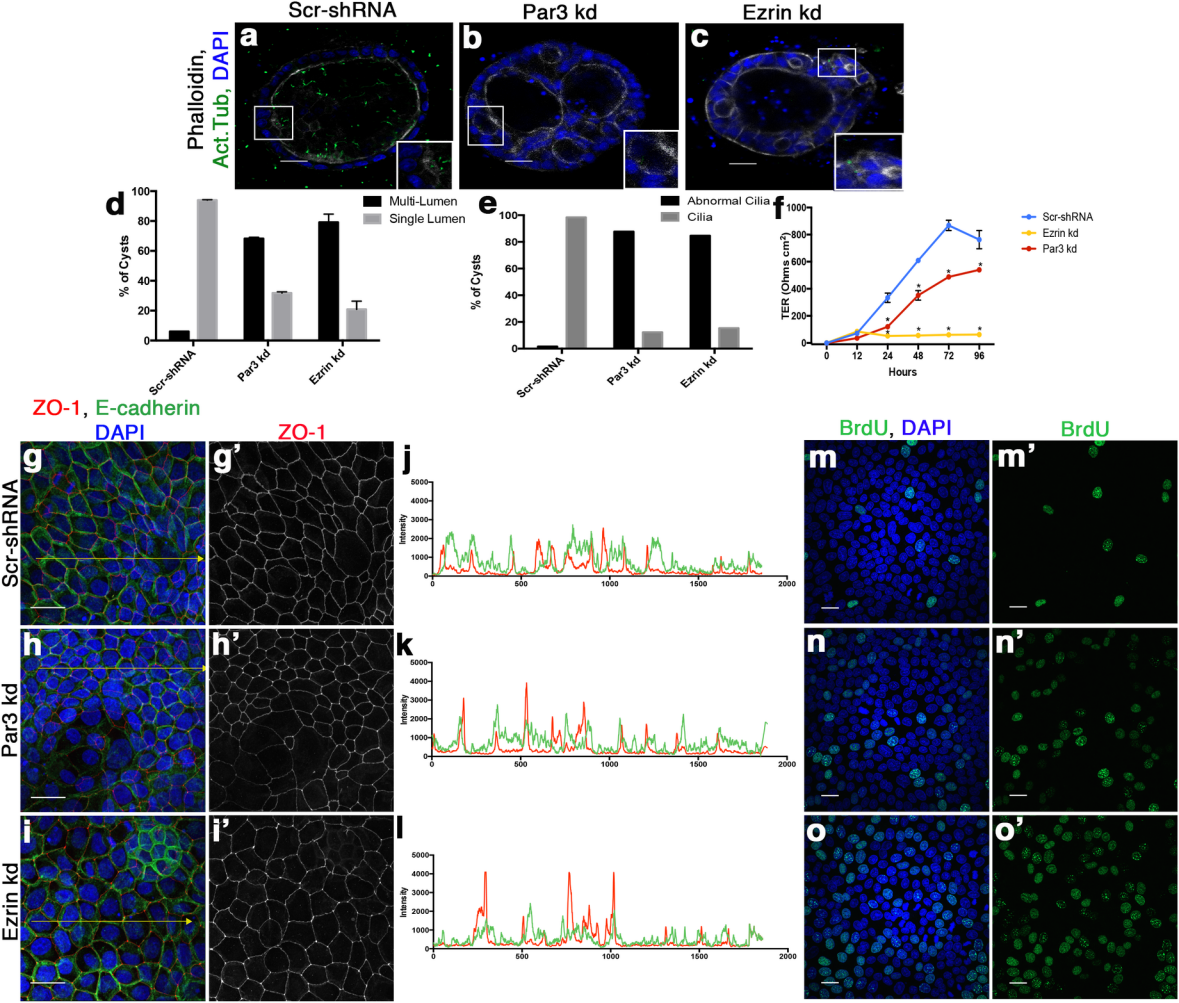
Depletion of apical polarity proteins cause a decrease in differentiation markers in epithelial 3D cell culture. (**a-c**) Immunofluorescence staining on (**a**) Scramble-shRNA (Scr-shRNA), (**b**) Par3-shRNA (Par3-kd), and (**c**) Ezrin-shRNA (Ezrin-kd) 3D cysts for primary cilia by acetylated tubulin (ac. tub) and actin by Phalloidin. Scale bar, 20 μm (**d, e**) Quantification of the (**d**) number of lumens (n = 2) and (**e**) cilia (n = 1) present within all 3D cysts compared to scramble control cells. Par3-kd, Ezrin-kd, and Scr-shRNA had at least 49 cysts, 13 cysts, or 70 cysts examined per independent experiment. Abnormal cilia include cysts without cilia present or cilia that appears abnormal. Error bars represent SEM. (**f**) Transepithelial resistance demonstrates loss of functional TJ in Par3-kd and Ezrin-kd cells compared to Scr-shRNA cells calculated by Ohms per cm^2^. (**g-i**) Immunofluorescence staining for ZO-1 shows altered TJ protein localization in Par3-kd and Ezrin-kd cells indicative of decrease in differentiation by loss of epithelial cell junctions. E-cadherin is also stained to label junctional complexes. Scale bar, 20 μm (**g’-i’**) ZO-1 only staining to show the aggregation of ZO-1 at tricellular junctions in white. (**j-l**) Line plots of (**g-**i) showing intensity of E-cadherin (green line) and ZO-1 (red line) on the yellow line in the respective image, Scr-shRNA (**j**), Par3-kd (**k**), and Ezrin-kd (**l**). Note the overlap in E-cadherin and ZO-1 intensities in Scr-shRNA compared to distinct peaks of ZO-1 intensity in Par3-kd and Ezrin-kd indicative of mislocalized ZO-1. (**m-o**) Increased BrdU incorporation observed in Par3-kd and Ezrin-kd cell lines compared to the Scr-shRNA cells. (**m’-o’**) BrdU only staining of (**m-o**). Scale bar, 20 μm.

To understand how apicobasal polarity was regulating differentiation we utilized a commonly used polarized epithelial cell model, Madin-Darby Canine Kidney (MDCK) cells, as we were unable to obtain and manipulate normal endometrial epithelial cells. Apicobasal polarity was disrupted through shRNA-mediated knockdown of either Par3 or Ezrin (S2 Fig). To better recapitulate the 3D organization of the endometrium, we cultured Scramble-shRNA (Scr-shRNA), Par3-shRNA (Par3-kd) and Ezrin-shRNA (Ezrin-kd) cells in a 3D matrix and stained the cells for acetylated tubulin, a marker of cilia, Phalloidin, E-cadherin or ZO-1 (Fig 3A–3C) (S2 Fig). Scr-shRNA cells formed single lumen structures with numerous cells extending cilia into the luminal space (Fig 3A). Confirming previous studies, we observed both Par3-kd and Ezrin-kd cells formed multi-lumen structures (Fig 3A–3C and 3D) [41,42] similar to what was observed in human EECs (Fig 1B and 1E) [43]. Additionally, we observed an overall decrease in the presence of cilia (Fig 3A–3C and 3E) analogous to our observations in endometrial cancer (S2 Fig). Both the multi-lumen phenotype and the loss of cilia are indicative of a less differentiated state in epithelial cells [38–40,43–45], implying that disruption of apicobasal polarity in the cell-based model caused a less differentiated state similar to EECs.

To provide additional evidence for altered differentiation in our Par3-kd and Ezrin-kd cell model, we examined the localization of the polarized tight junction (TJ) marker ZO-1 and the formation of functional TJs, a known sign of differentiation in MDCK cells [39]. The Par3-kd and Ezrin-kd cells displayed high concentrations of ZO-1 at tricellular contacts; however ZO-1 levels were low or absent at other apical contact points (Fig 3G–3I, 3G’–3I’ and 3J–3L). Similarly in 3D cultures ZO-1 had a disorganized staining pattern in the Par3-kd and Ezrin-kd cells compared to control cells where it localized to the apical junctional border (S2 Fig). Additionally, the Scr-shRNA cells show an increase in TER over time; by contrast, the Par3-kd, as previously described [35], and Ezrin-kd cell lines do not increase TER to the same degree (Fig 3F), indicating that these cells are less differentiated and cannot form a functional, polarized TJ. We also sought to determine whether disruption of apicobasal polarity increased cell proliferation, another marker of decreased cell differentiation. Cells depleted of either Par3 or Ezrin displayed increased BrdU incorporation compared to the control cells (Fig 3M–3O and 3M’–3O’) (S2 Fig). In concordance with our observations in low-grade endometrial cancer, these data demonstrate that disruption of apicobasal polarity decreases differentiation of epithelial cells.

### Disruption of apicobasal polarity decreases Notch signaling in epithelial cells and in low-grade endometrial cancer

**Fig 4:**
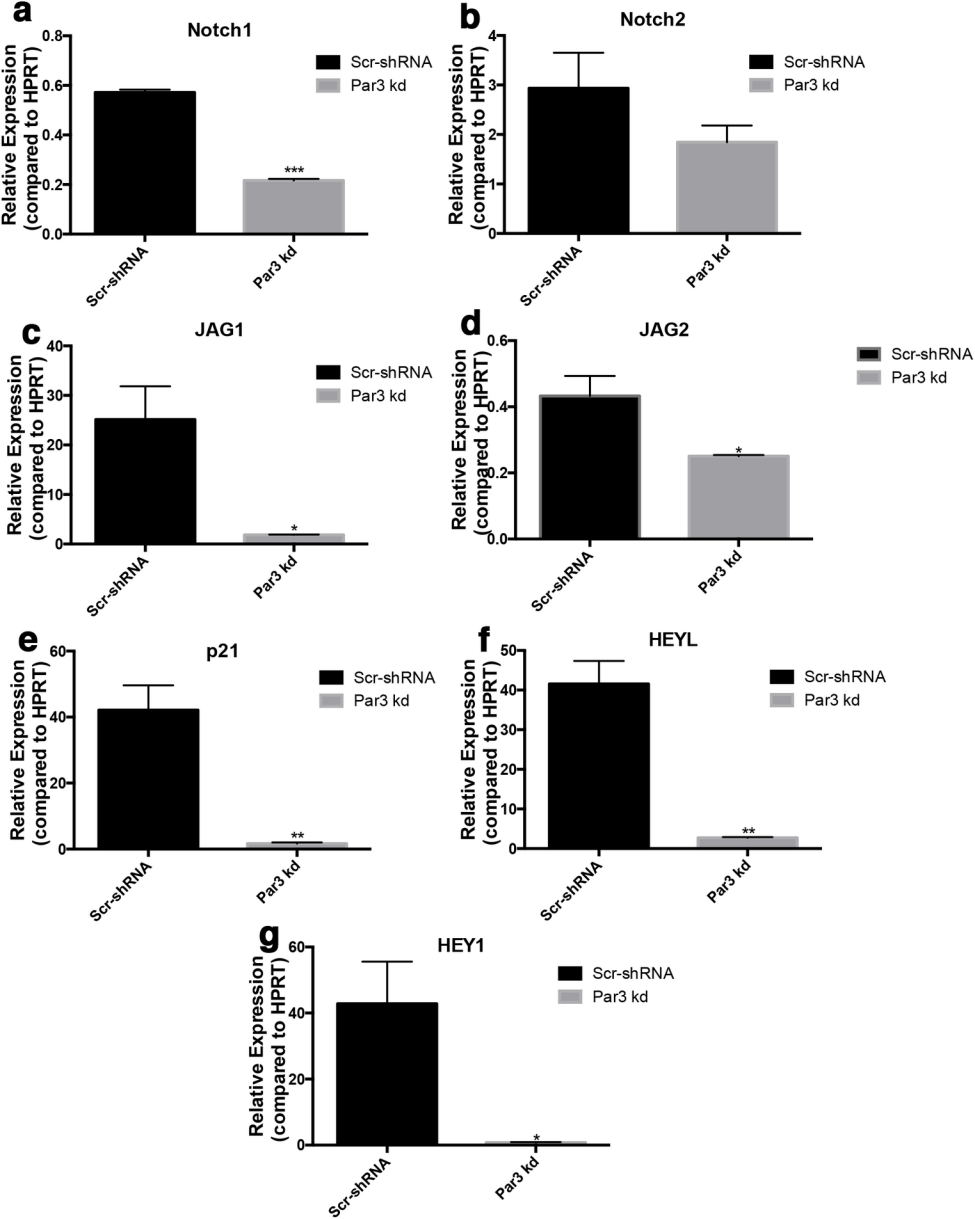
Notch signaling decreases in Par3 depleted epithelial cells. (**a-g**) qRT-PCR analysis of Notch1 (**a**), Notch2 (**b**), JAG1 (**c**), JAG2 (**d**), p21 (**e**), HEYL (**f**), or HEY1 (**g**) expression in Scr-shRNA cells and Par3-kd cells. Error bars signify SEM. * <0.05, ** <0.001, ***<0.0001.

To examine the underlying signaling pathways that could regulate differentiation in cells with disrupted polarity we utilized our Scr-shRNA, Par3-kd and Ezrin-kd MDCK cells. We examined Notch signaling as it regulates proliferation and differentiation in a tissue-specific manner *in vitro* and *in vivo* [46–49]. Notch signaling is tightly regulated in the normal endometrium during the menstrual cycle to assist in increasing proliferation and decreasing differentiation [22]. Furthermore, in *Drosophila* Notch receptor localization is affected by proteins known to regulate polarity [50]. Using qRT-PCR, we found MDCK cells express Notch1 and Notch2, and the Notch ligands, Jag1 and Jag2, along with several Notch targets known to play a role in differentiation and proliferation including HEYL, HEY1, and p21 (S3 Fig) [46–49]. We observed a significant decrease in the expression of Notch receptors (Fig 4A and 4B) (S3 Fig), Notch ligands (Fig 4C and 4D) (S3 Fig), and Notch downstream targets (Fig 4E–4G) (S3 Fig) in both Par3-kd and Ezrin-kd cells indicating that altered apicobasal polarity disrupts Notch signaling in this mammalian cell-based model.

**Fig 5:**
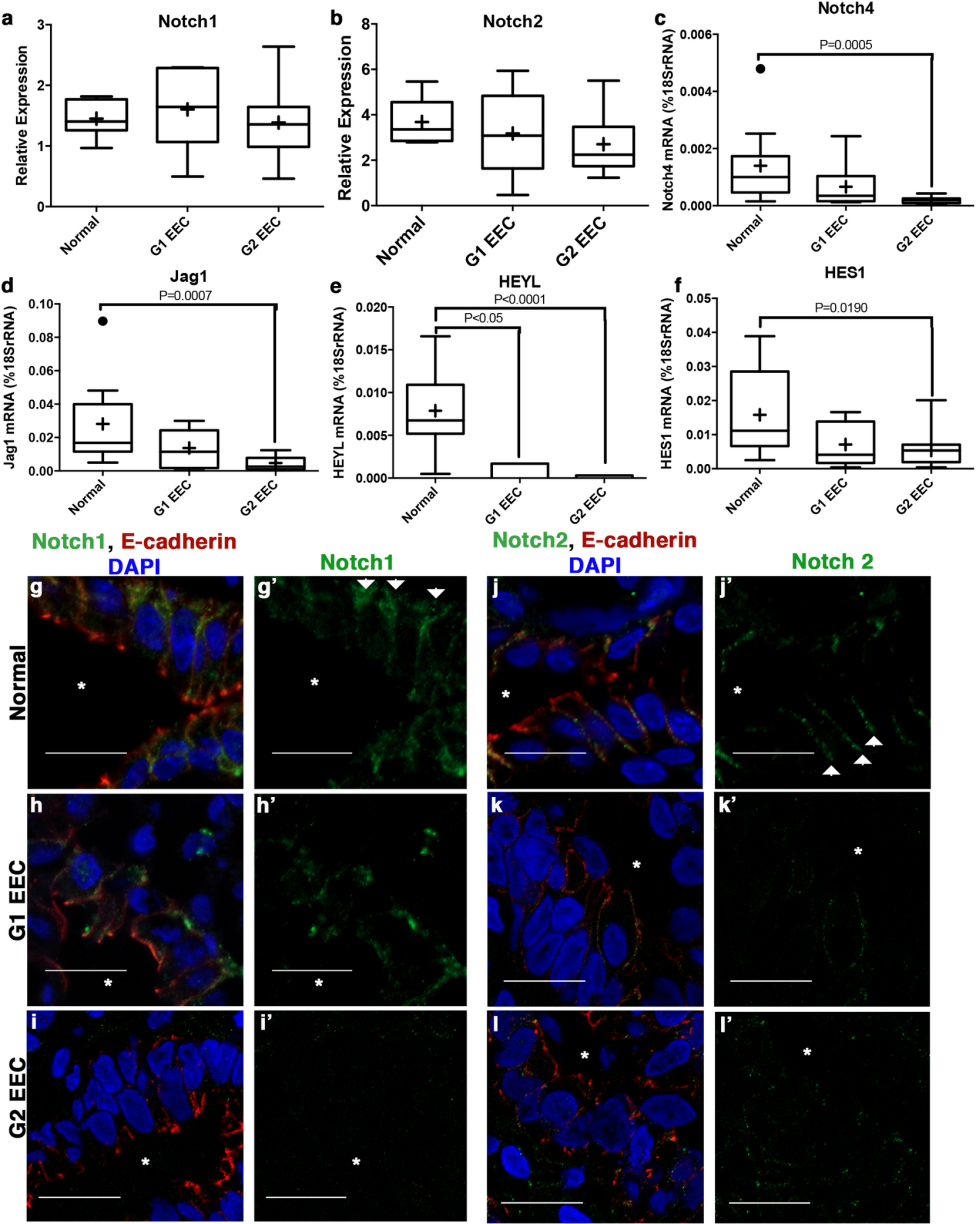
Notch downstream signaling and receptor localization is disrupted in low-grade endometrial cancer. (**a-f**) qRT-PCR of Notch receptors, ligands, and downstream targets in normal and in low-grade endometrial cancer (G1 & G2 EEC). The Notch receptor (**a**) Notch1 and (**b**) Notch2 show no change in expression while (**c**) Notch4 is decreased. Notch ligand (**d**) Jag1 and downstream targets (**e**) HEYL and (**f**) HES1 are down regulated in low-grade endometrial cancer. (**a**) Notch1 and (**b**) Notch2 data was analyzed using ΔCT with the Tata box binding protein (DBP) as the reference gene with 7 samples for Normal, G1 EEC, and G2 EEC. (**c-f**) Notch4, Jag1, HES1, and HEYL were analyzed by calculating the number of molecules of the gene of interest compared to 18SrRNA (%18SrRNA). Tukey box plots were used with SEM where + is the mean value and • are outliers. Normal (n = 10), G1 EEC (n = 9), and G2 EEC (n = 22). (**g-l**) Immunofluorescence of Notch receptors showing localization of (**g-i, g’-i’**) Notch1 and (**j-l, j’-l’**) Notch2 in (**g, j**) normal endometrium, (**h, k**) G1 EEC, and (**i, l**) G2 EEC. E-cadherin marks the basolateral cell:cell contacts (g-l) (*) signifies the lumen. Scale bar, 20 μm. (g’-l’) Images of (g-l) with Notch1 or Notch2 respectively showing localization to the lumen (*). Arrows denote lateral localization of Notch1 or Notch2.

We next asked whether a similar change occurs in the Notch signaling pathway in low-grade EECs. We performed qRT-PCR with normal endometrium, G1, and G2 EEC samples. We observed a significant decrease in the Notch downstream targets, HEYL and HES1 (Fig 5E and 5F) and the Notch ligand, Jag1 (Fig 5D), indicating that overall Notch signaling is decreased in low-grade EEC. Additionally, we detected a significant decrease in the transcript levels of the Notch receptor, Notch4 but no decrease in Notch1 or Notch2 (Fig 5A–5C). Previous work has demonstrated that overall levels of active Notch intracellular domain is critical for downstream Notch signaling suggesting that Notch1 and/or Notch2 protein could be regulated in other manners in low-grade EEC [32,51].

Our observation that Notch1 and Notch2 mRNA expression levels were similar, but that downstream targets were reduced, in normal compared to EEC samples, prompted us to examine the Notch1 and Notch2 proteins. The Notch receptors are membrane-bound proteins whose activation may be affected by subcellular localization [52]. In normal human endometrium Notch1 and Notch2 localize to the basolateral and lateral membranes of the glandular epithelial cells, respectively (Fig 5G, 5J, 5G’ and 5J’). In low-grade EECs, by contrast, neither Notch1 nor Notch2 receptor proteins localize correctly to the lateral membrane of epithelial cells. Moreover, Notch1 and Notch2 protein levels are decreased in the tumor (Fig 5H, 5I, 5K and 5L). Collectively, these data show that low-grade EECs have disrupted apicobasal polarity and mislocalized and/or decreased protein levels of Notch1 and Notch2 receptors that may lead to the overall decrease observed in Notch downstream targets.

### Expression of Par3 in endometrial cancer cells promotes differentiation and decreased proliferation

**Fig 6:**
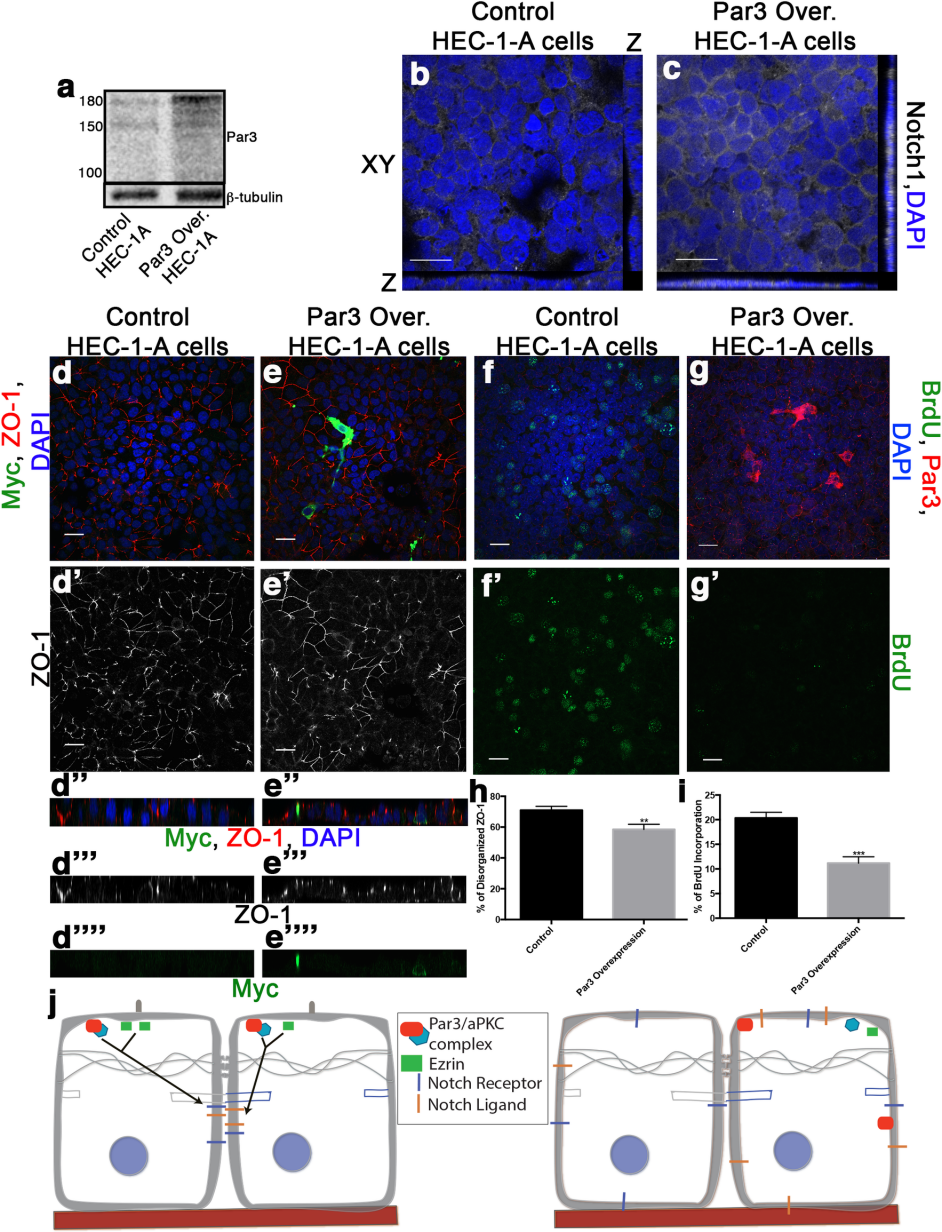
Expressing Par3 in endometrial cancer cell lines blocks proliferation and promotes differentiation. **(a)** Western blot analysis of Par3 in control transfection (control) and Myc-Par3 overexpression Hec-1-A cells. (**b,c**) Parental Hec-1-A or Hec-1-A with exogenous Par3 stained with DAPI and Notch1. (**d-g, d’-g’**) Staining of parental (**d, f**) and Par3 overexpressing (**e, g**) Hec-1-A cells. Cells were stained for Myc-Par3, ZO-1, and DAPI (**d,e**) or Par3, BrdU, and DAPI **(f, g).** Scale bar, 20 μM. (**d’, e’**) ZO-1 only staining (**d”-d””, e”-e””**) Z-plane showing ZO-1, Myc and/or DAPI staining. (**f’, g’**) Images of **(f,g)** with BrdU staining only. (**h**) Quantification of disorganized ZO-1 in the parental (n = 3) and Par3 overexpression Hec-1-A cells (n = 3) for at least three regions of interest (ROI) per experiment. Error bars represent SEM *<0.05. (**i**) Quantification of BrdU incorporation in the parental (n = 3) and Par3 overexpression Hec-1-A (n = 3) cells for at least three ROI per experiment. Error bars represent SEM *0.05. (**j**) Schematic of proposed model for how apicobasal polarity controls differentiation of endometrial epithelial cells by regulating Notch receptor localization and, Notch downstream targets that modulate proliferation and differentiation.

To determine whether establishment of apicobasal polarity alters differentiation or proliferation in endometrial cancer, we assayed endometrial cancer cell lines for changes in apical polarity proteins. We examined Par3 expression in a panel of endometrial cancer cell lines from G1, G2, and G3 tumors [19]. Western blot analysis revealed that Par3 was not readily detected in a majority of the endometrial cancer cell lines (S4 Fig). To determine how tumor cells would respond to Par3 expression, we overexpressed Par3 in Ishikawa and Hec-1-A cells (Fig 6A) (S4 Fig). In contrast to parental cells lacking Par3, in which the TJ protein ZO-1 did not localize to apical cell contacts in an organized pattern, we observed a more fence-like organization of ZO-1 in cells overexpressing Par3 (Fig 6D–6D””and 6E–6E””) (S4 Fig). These data are concordant with our previous observations (Fig 3) indicating Par3 expression can promote differentiation in endometrial tumor cell lines. Additionally, cells expressing exogenous Par3 showed an increase in Notch1 localization to cell:cell contacts compared to cytoplasmic Notch1 localization in parental cells with reduced levels of Par3 (Fig 6B and 6C). Finally, cells ectopically expressing Par3 showed lower levels of proliferation than in the parental endometrial tumor cell lines lacking Par3, as visualized by BrdU incorporation (Fig 6F, 6G and 6L) (S4F and S4H Fig). These data provide evidence that expression and apical localization of Par3 is critical for the proper differentiation of the endometrial epithelial cells, and that disruption of apicobasal polarity affects the ability of endometrial epithelium to regulate differentiation and proliferation (Fig 6J).

### Decrease in proliferation and migration observed in Par3 overexpressing cells through Notch signaling

**Fig 7:**
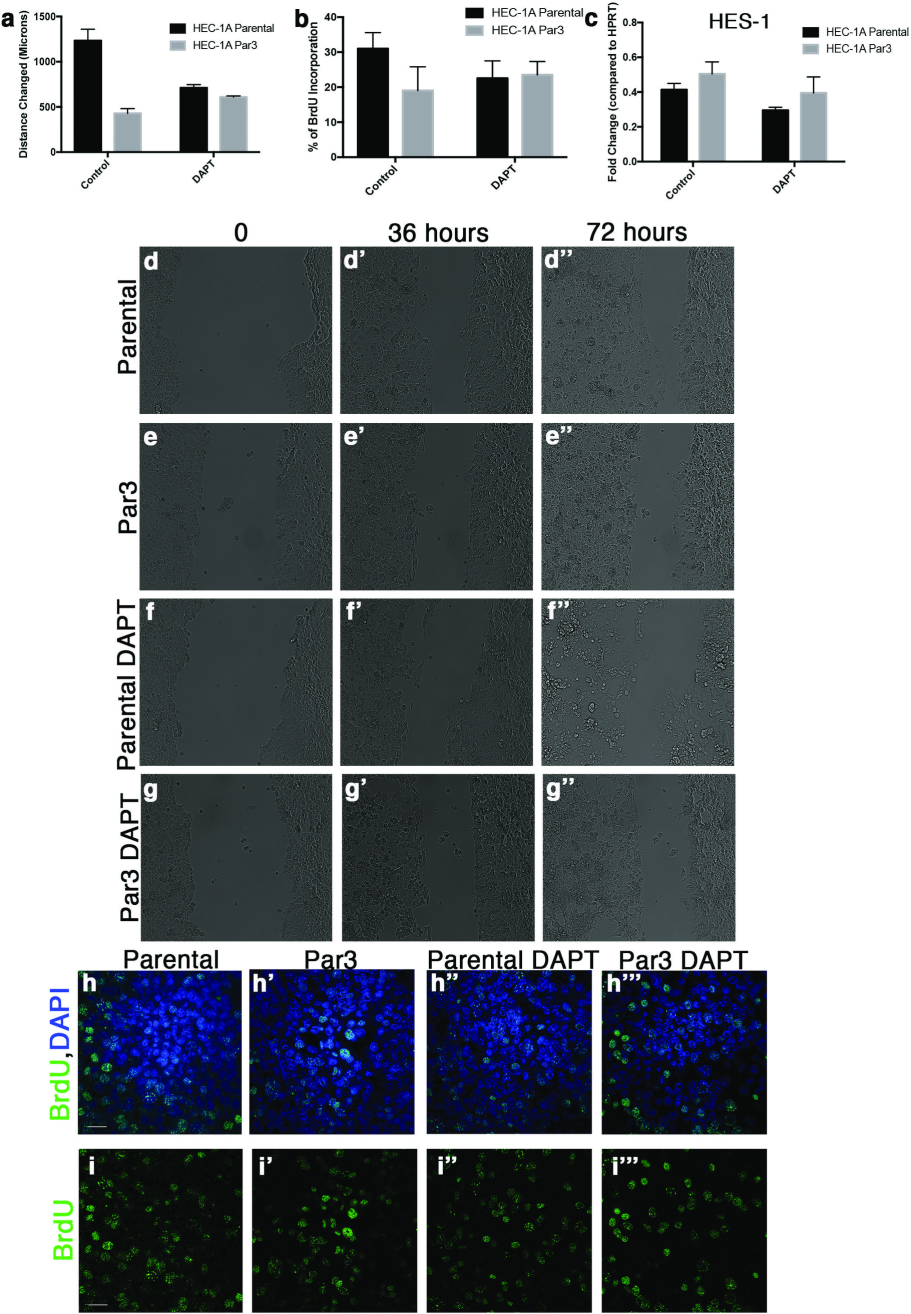
Inhibiting Notch signaling rescues Par3 mediated changes in migration and proliferation. (**a**) Quantification of cell migration for parental Hec-1-A cells, Par3 overexpression Hec-1-A cells, and Hec-1-A cells treated the gamma secretase inhibitor (DAPT) to block Notch signaling. (**b**) Quantification of BrdU incorporation in the parental, Par3 overexpression, and DAPT treated Hec-1-A cells. (**c**) qRT-PCR analysis of the Notch target HES-1 in parental, Par3 overexpression and DAPT treated Hec-1-A cells. (**d-g**) Photos showing specific times during the migration assay performed to examine rate of migration for Hec-1-A parental cells (**d-d”**), Hec-1-A with Par3 expression (**e-e”**), Hec-1-A parental cells treated with DAPT (**f-f”**), and Hec-1-A Par3 expressing cells treated with DAPT (**g-g”**). Immunofluorescence analysis of BrdU incorporation in parental Hec-1-A cells (**h, i**), Hec-1-A overexpressing Par3 cells (**h’, i’**), parental cells treated with DAPT (**h”, i”**) or Par3 expressing cells treated with DAPT (**h’”, i’”**). Top panels (**h-h’”**) show BrdU (green) with DAPI (blue) staining and panels (**i-i’”**) show BrdU staining alone. Scale bar, 20 μM.

Increased migration is a hallmark of metastatic cancer cells so migration was examined in the cultured endometrial cancer cell lines in the presence or absence of Par3. Par3 overexpression was found to decrease the rate of migration in Hec-1-A cells (Fig 7A, 7D, 7E, 7D’, 7E’, 7D”and 7E”). In addition, a similar trend was observed in Ishikawa cells, where Par3 overexpression decreased the distance that cells moved. (S5 Fig). To determined if the changes to proliferation and migration were related to Notch signaling, we utilized the gamma-secretase inhibitor N-[N-(3,5-Difluorophenacetyl)-L-alanyl]-S-phenylglycine t-butyl ester (DAPT). Cells overexpressing Par3 were treated with DAPT and the rate of migration and proliferation were measured. We determined Par3 expressing cells treated with DAPT had similar amounts of migration as either Hec-1-A or Ishikawa parental cell lines respectively treated with DAPT (Fig 7A, 7F, 7G, 7F’, 7G’, 7F” and 7G”; S5 Fig). Additionally, BrdU incorporation was similar between parental cell lines and Par3 overexpression cell lines when treated with DAPT (Fig 7B, 7H”, 7I”, 7H”‘ and 7I”‘; S5 Fig). Our observations that DAPT treatment caused Par3 overexpression cell lines to migrate and proliferate similar to the parental cell lines led us to examine the Notch signaling downstream target, HES-1 by qRT-PCR. HES-1 was found to increase when Par3 was overexpressed in either Hec-1-A or Ishikawa cells (Fig 7C, S5 Fig). In addition, when cells were treated with DAPT there was a decrease in HES-1 in both parental and Par3 overexpression cell lines demonstrating that Notch signaling was blocked in both cell lines (Fig 7C, S5 Fig). These results demonstrate that Par3 expression increases Notch signaling in endometrial cancer cell lines resulting in decreased proliferation and migration.

## Discussion

This study shows that loss of apicobasal polarity in low-grade endometrial cancer is independent of the loss of adherens junctions. While previous studies have shown that Par3 is important in breast cancer metastasis [7] and that loss of the von Hippel-Lindau tumor suppressor protein, a known regulator of polarity proteins, is associated with increased risk for numerous cancer types [53–56], little is known about what role polarity has in the early stages of cancer. Our data indicate that loss of apicobasal polarity may promote the progressive decrease in signaling and cellular differentiation found in EEC. Low-grade EEC is frequently associated with early stage cancer; accordingly, our data suggest that disruption of apicobasal polarity plays a role in regulating differentiation, proliferation and migration in early endometrial cancer cells. While this work does not define loss of Par3 as an initiating event in EEC it does demonstrate that polarity regulates many pathways associated with low-grade EEC including changes in proliferation and differentiation. In addition to EEC it will be interesting to determine if pre-cancerous lesions and hyperplasia of the endometrium have disrupted polarity or if altered polarity is a hallmark of EEC.

In addition to disrupted polarity, we also observed mislocalization of Notch receptors in low-grade endometrial cancers as well as decreases in Notch target gene expression in low-grade EECs. Many Notch downstream targets are critical for differentiation and proliferation in multiple tissue types, but the role of the Notch pathway in endometrial cancer remains unclear [22,46–49,57–59]. Previous studies have linked disruption of the Akt/PTEN pathway, one of the most common pathways altered in endometrial cancer, with aberrant Notch signaling in prostate cancer [60,61]. Our findings of significant down regulation in Notch signaling in endometrial cancer might indicate a similar cooperation between the Notch pathway and an increase in the Akt pathway commonly observed in endometrial cancer [20]. Additionally, the decreased Notch pathway signaling in endometrial cancer may promote the progression of tumors. Because the loss of polarity causes a decrease in Notch signaling in epithelial cells, we postulate that establishment of apicobasal polarity is critical for normal differentiation and proliferation through signaling pathways such as Notch. Indeed, disruption of polarity in epithelial cells caused a significant decrease in phenotypes associated with changes in cellular differentiation, specifically TJ formation, the presence of cilia and a decrease in overall proliferation. These changes in proliferation and differentiation are similar to changes observed in CNS stem cells following alterations to Notch signaling [32]. Furthermore, following overexpression of Par3, endometrial cancer cell lines obtain markers indicative of a more differentiated state, including TJ protein organization, decreased cell packing and a decrease in proliferation. Additionally, overexpression of Par3 in the endometrial cancer lines increased Notch signaling in turn decreasing proliferation and migration that can be reversed by treating cells with the gamma-secretase inhibitor DAPT. Interestingly, overexpression of Par3 in endometrial cancer cell lines appears to produce both cell autonomous and cell non-autonomous effects. The possible cell non-autonomous effect was observed when the cells that show no or low amounts of Par3 display decreased proliferation and increased differentiation phenotypes similar to the cells ectopically expressing Par3. This implies that disruptions in cell polarity in one tumor cell could have deleterious effects on the neighboring normal cells, thereby providing an environment conducive to tumor growth and proliferation. Similar to our results, others have reported both cell autonomous and cell non-autonomous changes in signaling pathways regulating proliferation when basal polarity or AJ proteins are depleted, suggesting that cell intrinsic changes in polarity and/or AJ-mediated adhesion can be conveyed over a local area of the tissue [62].

Our data demonstrate that apicobasal polarity is disrupted in low-grade endometrial cancer prior to disruption of cell adhesion. This was unexpected given previous reports linking disruption of apicobasal polarity to loss of cell adhesion in cancer [63]. Disruption of apicobasal polarity results in a less differentiated and more proliferative state in epithelial cells while exogenous expression of Par3 within endometrial cancer cell lines promotes differentiation and inhibits proliferation. We also provide data showing that disruption of polarity causes a decrease in Notch signaling *in vitro*, similar to observations in endometrial cancer. The decrease in Notch signaling, along with the mislocalization of Notch receptors, implies that polarity is critical for proper membrane partitioning of Notch receptors in the endometrium. The ability of polarity protein disruption to affect epithelial differentiation and proliferation may be at least partially mediated through the Notch pathway, specifically through localization of Notch receptors and ligands. The dynamic nature of endometrial tissue demands accurate regulation of apicobasal polarity in endometrial reorganization. Future studies examining how apicobasal polarity and/or downstream signaling pathways can be manipulated in dynamic tissues such as the endometrium could lead to better therapeutic strategies for patients with endometrial cancer, thus resulting in improved future overall health for the patient.

## Supporting information

S1 Table List of primers used for qRT-PCR analysis. List of primers sequences used for qRT-PCR analysis of Notch pathway components.
(PDF)

S1 Fig Loss of cilia in endometrial tumors with disrupted apicobasal polarity. Corresponds to Fig 2.
Staining with antibodies against acetylated tubulin (Ac. Tub), a marker of cilia, pan cytokeratin (Pan Cyto.), an epithelial marker, and DAPI in (**a**) normal endometrium, (**b**) G1 EEC and (**c**) G2 EEC shows a decrease in cilia indicative of decreased differentiation. Scale bar, 20 μm. (**d**) Quantification of the number of cilia found per gland determined from 10 lumens of each sample (n = 3 normal, n = 5 EEC). Error bars represent SEM.
(TIF)

S2 Fig Depletion of polarity proteins causes an increase in multiple lumen structures in epithelial 3D cell culture. Corresponds to Fig 3.
(**a, b**) Western blot analysis of (**a**) Par3 or (**b**) Ezrin knockdown in the MDCK cells compared to a scramble control. (**c-e**) Orthogonal view of (**c**) scr-shRNA, (**d**) Ezrin-shRNA or (**e**) Par3-shRNA with E-cadherin (green), ZO-1 (red), and DAPI showing multiple lumens in cysts depleted of apical polarity proteins. (**f**) Quantification of the number of BrdU positive cells in Fig 3M’–3O’.
(TIF)

S3 Fig Notch signaling receptors, ligands, and downstream targets expressed in MDCK epithelial cells. Corresponds to Fig 4.
(**a-c**) qRT-PCR analysis showing (**a**) Notch receptors, (**b**) Notch ligands, and (**c**) Notch downstream targets that are expressed in wild-type MDCK cells. Samples were done in triplicate.
(TIF)

S4 Fig Expressing Par3 in low-grade endometrial cancer cell lines causes differentiation phenotypes. Corresponds to Fig 6.
(**a**) Western blot analysis of a panel of endometrial cancer cell lines (HEC-1-B, HEC-1-A, Ishikawa, ECC-1, HEC-50, MFE-280, and MFE-296) for Par3 and E-cadherin. Ishikawa and ECC-1 are well-differentiated cell lines, HEC-1-A, HEC-1-B, MFE-296 are moderately differentiated cell lines, and HEC-50, MFE-280 are poorly differentiated cell lines. (**b**) Western blot analysis of Par3 in Ishikawa cells with and without exogenous Par3. (**c, d**) Staining of parental Ishikawa cells (**c**) and cells with exogenous Par3 (**d**) for Par3 (red), ZO-1 (green), and DAPI. (**c”- c”“, d”-d”“**) Z-plane showing ZO-1 apical-lateral localization to the junctions. Scale bar, 20μM. (**g)** Quantification of disorganized ZO-1 in the control (n = 3) and Par3 overexpression Ishikawa cells (n = 3) for at least 3 fields of view per experiment. Error bars represent SEM *<0.05. (**h**) Quantification of BrdU incorporation in the control (n = 3) and Par3 overexpression Ishikawa (n = 3) cells for at least 3 fields of view per experiment. Error bars represent SEM. *<0.05.
(TIF)

S5 Fig Inhibiting Notch in Ishikawa cells expressing Par3 reverses changes in migration and proliferation. Corresponds to Fig 7.
(**a**) Quantification of cell migration for parental Ishikawa cells, Par3 overexpression Ishikawa cells, and Ishikawa cells treated with DAPT. (**b**) Quantification of BrdU incorporation in the parental, Par3 overexpression, and DAPT treated Ishikawa cells. (**c**) qRT-PCR analysis of the Notch target HES-1 in parental, Par3 overexpression and DAPT treated Ishikawa cells. (**d-g**) Photos showing specific times during the migration assay to examine rate of migration for Ishikawa parental cells (**d-d”**), Ishikawa cells with Par3 expression (**e-e”**), Ishikawa parental cells treated with DAPT (**f-f”**), and Ishikawa Par3 expressing cells treated with DAPT (**g-g”**). Immunofluorescence analysis of BrdU in parental Ishikawa cells (**h, h’**), Ishikawa cells overexpressing Par3 (**i, i’**), parental cells treated with DAPT (**j, j’**) or Par3 expressing cells treated with DAPT (**k, k’**). Top panels (**h-k**) show BrdU (green) with DAPI (blue) staining and panels (**h’-k’**) show BrdU staining alone. Scale bar, 20 μM.
(TIF)

S1 Dataset Individual data points files. Spreadsheet providing individual data points for the data acquired in the manuscript. Data points are divided between specific figures on distinct tabs.
(XLSX)
