# Supplementary material for: Loss of polarity alters proliferation and differentiation in low-grade endometrial cancers by disrupting Notch signaling
Source: PLoS One. 2017 Dec 5;12(12):e0189081. doi: 10.1371/journal.pone.0189081 (PMC5716545; doi:10.1371/journal.pone.0189081)
Supplement: S1 Table — List of primers sequences used for qRT-PCR analysis of Notch pathway components. (PDF) [file pone.0189081.s001.pdf]

Supplementary Table 1. Primers utilized for quantitative RT-PCR

Name	R/F	Sequence
HPRT	F<br>R	AGCTTGCTGGTGAAAAGGAC<br>TTATAGTCAAGGGCATATCC
Notch1 -1	F<br>R	CAGCCCTTGTCTCCAGAATG<br>TTGGCACCGTTCTTACAGG
Notch1 -2	F<br>R	GGATGGCATCAATAGCTTCATG<br>CCAGGGTCACAGTCACATTTG
Notch2 -1	F<br>R	TCGGGATAGCTATGAGCCCT<br>GGCATGTTGCTTTCCCCAAC
Notch2 -2	F<br>R	ATTTCATGCAGGTTAGAGAAGGAC<br>CTGTCTGAGAGCTCAGTGACCTTA
Notch3 -1	F<br>R	ATCAACCGCTATGACTGTGTC<br>TCCATTTTCCCCATCCACG
Notch3 -2	F<br>R	GCTGCGAAACTGATGTCAAC<br>GCTACTCTGACACTCATCCATG
Notch4 -1	F<br>R	AGTAACCCCTCAAACACAGC<br>ACAAATCCACACCCATCACC
Notch4 -2	F<br>R	GGTAAACCCATGTGAGTCCAG<br>AGTTCTGTCCATTGTAGCCTG
JAG1 -1	F<br>R	GAAAGTGCCCAGAGCCTAAA<br>CAGGACAGCTGAAGAACTGAA
JAG1 -2	F<br>R	GAACCTGATTGCGAGCTACTAC<br>GGTGGACAGATACAGCGATAAC
JAG2 -1	F<br>R	GGACGCCAATGAATGTGAAG<br>CGGGATGCAGAGACAGTAATAG
JAG2 -2	F<br>R	ACCTGATTGGTGGCTATTACTG<br>CTGCCCATGACAGTCGTTTA
DLL1 -1	F<br>R	CCGATGACCTCACAACAGAAA<br>CACACGAAGCGGTAGGAATAC
DLL1 -2	F<br>R	GCAGATCAAGAACACCAACAAG<br>GTCCAAAGGACAGCAAGA
DLL3 -1	F<br>R	GAATCACCCTGAAGATGGAGAC<br>GCTCCAAAGGACAGCAAGA
DLL3 -2	F<br>R	CGGATGGACCTTGCTTCAAT<br>ACAGTTGGAGCCTTGGAATC
DLL4 -1	F<br>R	GCTCAAGAACACAAACCAGAAG<br>GGGCCAGATTATAGTCCAATGT
DLL4 -2	F<br>R	TCCACTGGCATCTGTGTTTC<br>CCTCCTCTCTCCTCTCTGATTT
P21 -1	F<br>R	CATCCTGGCCTGTACTGTT<br>CTTGCCCTTCAGAGGCTTATAG
P21 -2	F<br>R	AGCTGAACAAGGAGTCAGATG<br>CAGGGCCAGAAGAGACAATAA
HeyL -1	F<br>R	AGTTGATCTTGGGTTCACTCTC<br>ACCAGAAAGGCTTGGGAATAG
HeyL -2	F<br>R	GTTCTTCATCCAGGGAGCTCTAAA<br>GAGGAAGATGCCTTCACAGATAG

**Hey1 -1**	**F**<br>**R**	**GCTATGGACTATCGGAGTTTGG**<br>**CTGGGAGGCGTAGTTGTTAAG**
**Hey1 -2**	**F**<br>**R**	**GCCCGATATCTGAGCATCATT**<br>**CGTAGTTGTTAAGGTGGGAGAC**
**Hey2 -1**	**F**<br>**R**	**GCGGCGAGATCGGATAAATAA**<br>**TGATCTACCGTCATTTGCAGTAT**
**Hey2 -2**	**F**<br>**R**	**AACATCTCAGATTATGGCAAGAAAG**<br>**CCAGTCGTCTCAACTCAGATAAA**
**Hes6 -1**	**F**<br>**R**	**AAAGCGCGCCTAGACAAA**<br>**CCGTCTGGTCTTGTAACTTGAT**
**Hes6 -2**	**F**<br>**R**	**CCAACACCTGTCGCTCTT**<br>**CTTGTAACTTGATGCCCATGAC**
