# Supplementary figures and images for: Loss of polarity alters proliferation and differentiation in low-grade endometrial cancers by disrupting Notch signaling
Source: PLoS One. 2017 Dec 5;12(12):e0189081. doi: 10.1371/journal.pone.0189081 (PMC5716545; doi:10.1371/journal.pone.0189081)

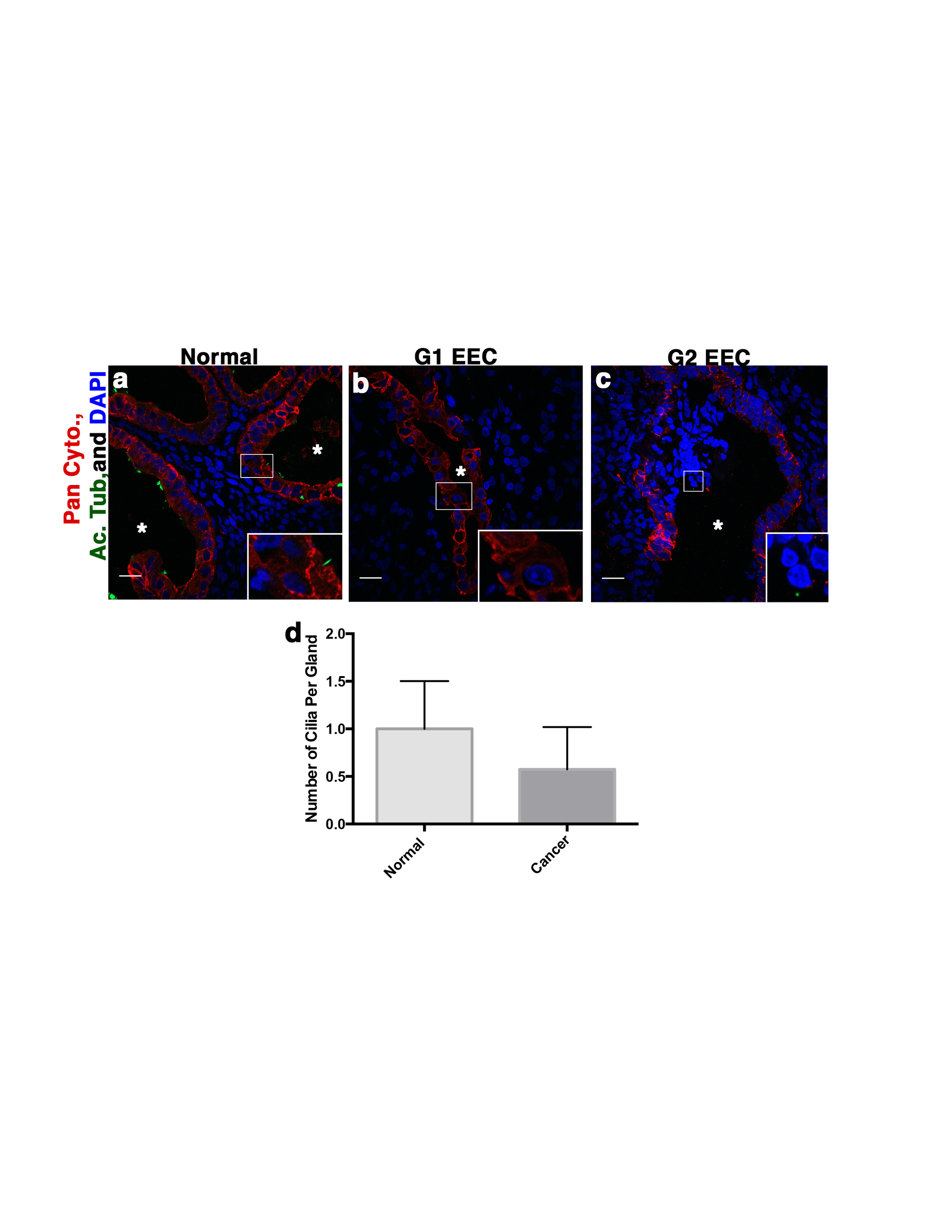

Supplement: S1 Fig — Corresponds to Fig 2. Staining with antibodies against acetylated tubulin (Ac. Tub), a marker of cilia, pan cytokeratin (Pan Cyto.), an epithelial marker, and DAPI in (a) normal endometrium, (b) G1 EEC and (c) G2 EEC shows a decrease in cilia indicative of decreased differentiation. Scale bar, 20 μm. (d) Quantification of the number of cilia found per gland determined from 10 lumens of each sample (n = 3 normal, n = 5 EEC). Error bars represent SEM. (TIF) [file pone.0189081.s002.tif]

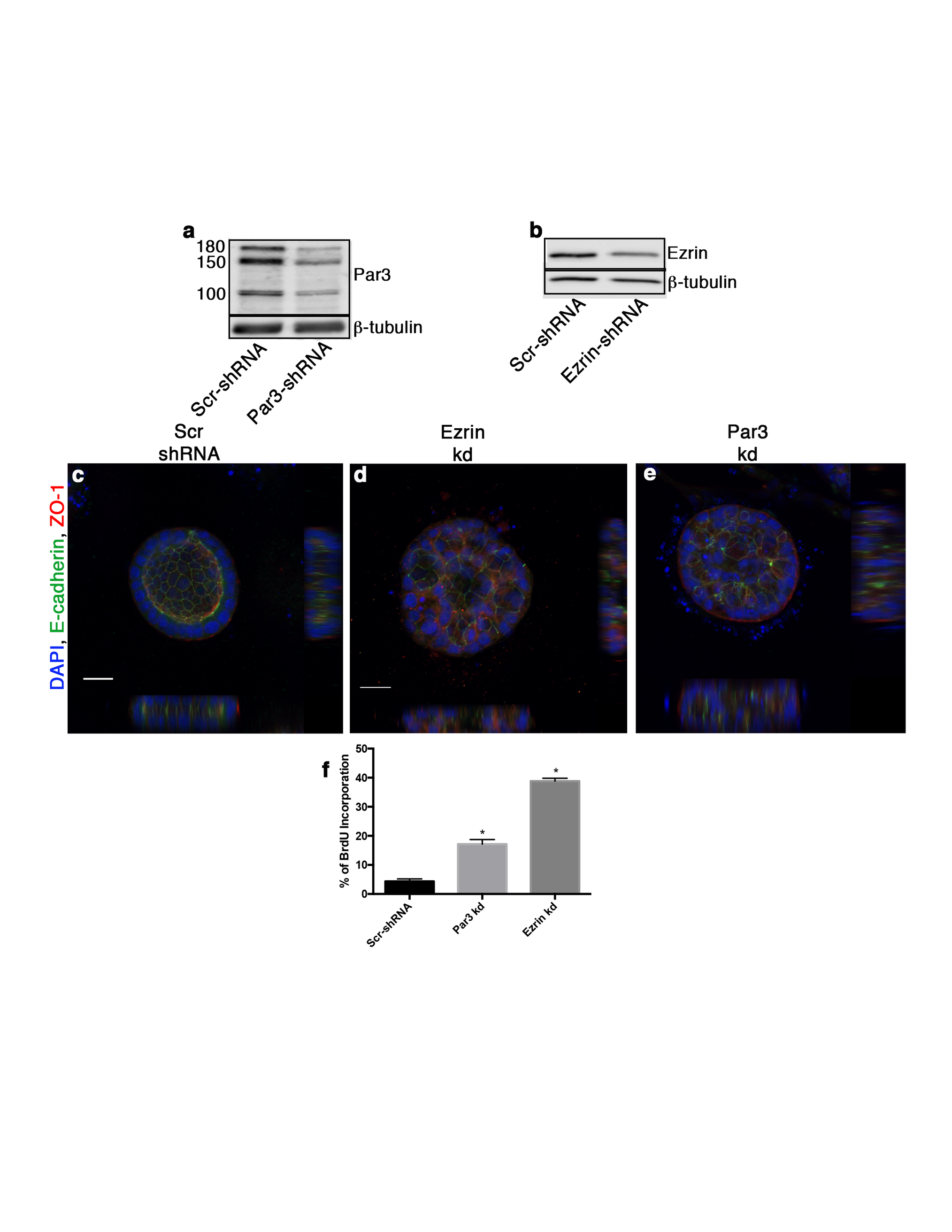

Supplement: S2 Fig — Corresponds to Fig 3. (a, b) Western blot analysis of (a) Par3 or (b) Ezrin knockdown in the MDCK cells compared to a scramble control. (c-e) Orthogonal view of (c) scr-shRNA, (d) Ezrin-shRNA or (e) Par3-shRNA with E-cadherin (green), ZO-1 (red), and DAPI showing multiple lumens in cysts depleted of apical polarity proteins. (f) Quantification of the number of BrdU positive cells in Fig 3M’–3O’. (TIF) [file pone.0189081.s003.tif]

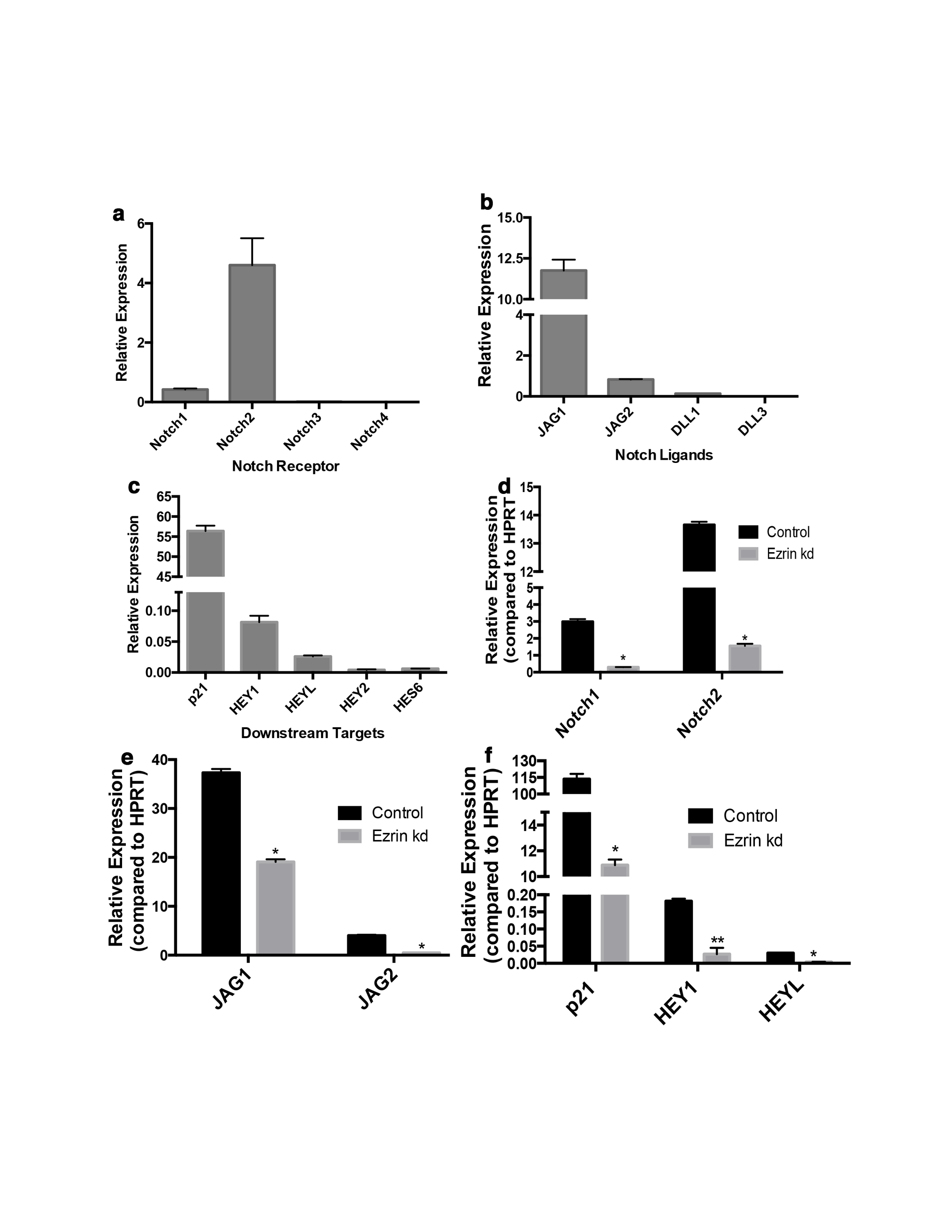

Supplement: S3 Fig — Corresponds to Fig 4. (a-c) qRT-PCR analysis showing (a) Notch receptors, (b) Notch ligands, and (c) Notch downstream targets that are expressed in wild-type MDCK cells. Samples were done in triplicate. (TIF) [file pone.0189081.s004.tif]

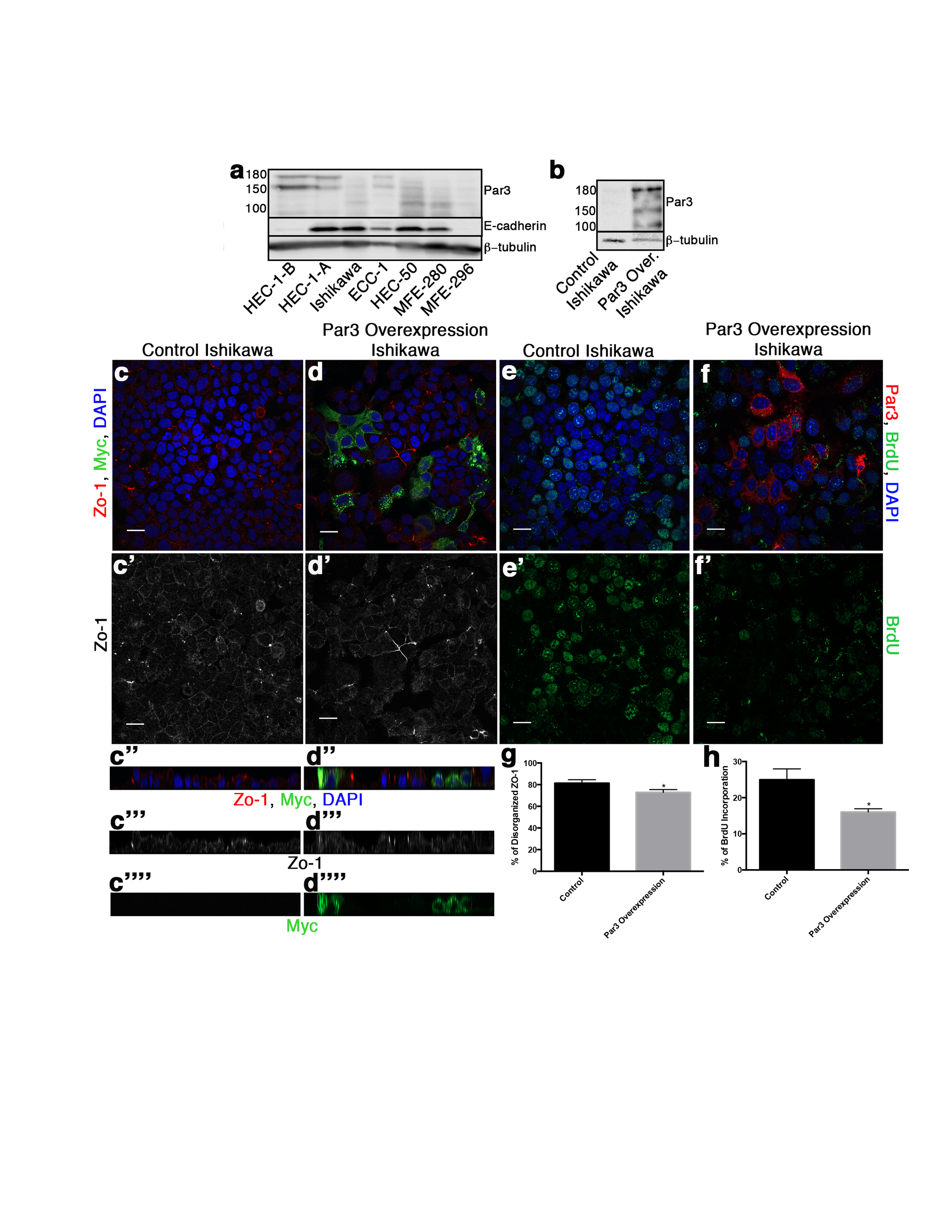

Supplement: S4 Fig — Corresponds to Fig 6. (a) Western blot analysis of a panel of endometrial cancer cell lines (HEC-1-B, HEC-1-A, Ishikawa, ECC-1, HEC-50, MFE-280, and MFE-296) for Par3 and E-cadherin. Ishikawa and ECC-1 are well-differentiated cell lines, HEC-1-A, HEC-1-B, MFE-296 are moderately differentiated cell lines, and HEC-50, MFE-280 are poorly differentiated cell lines. (b) Western blot analysis of Par3 in Ishikawa cells with and without exogenous Par3. (c, d) Staining of parental Ishikawa cells (c) and cells with exogenous Par3 (d) for Par3 (red), ZO-1 (green), and DAPI. (c”- c”“, d”-d”“) Z-plane showing ZO-1 apical-lateral localization to the junctions. Scale bar, 20μM. (g) Quantification of disorganized ZO-1 in the control (n = 3) and Par3 overexpression Ishikawa cells (n = 3) for at least 3 fields of view per experiment. Error bars represent SEM *<0.05. (h) Quantification of BrdU incorporation in the control (n = 3) and Par3 overexpression Ishikawa (n = 3) cells for at least 3 fields of view per experiment. Error bars represent SEM. *<0.05. (TIF) [file pone.0189081.s005.tif]

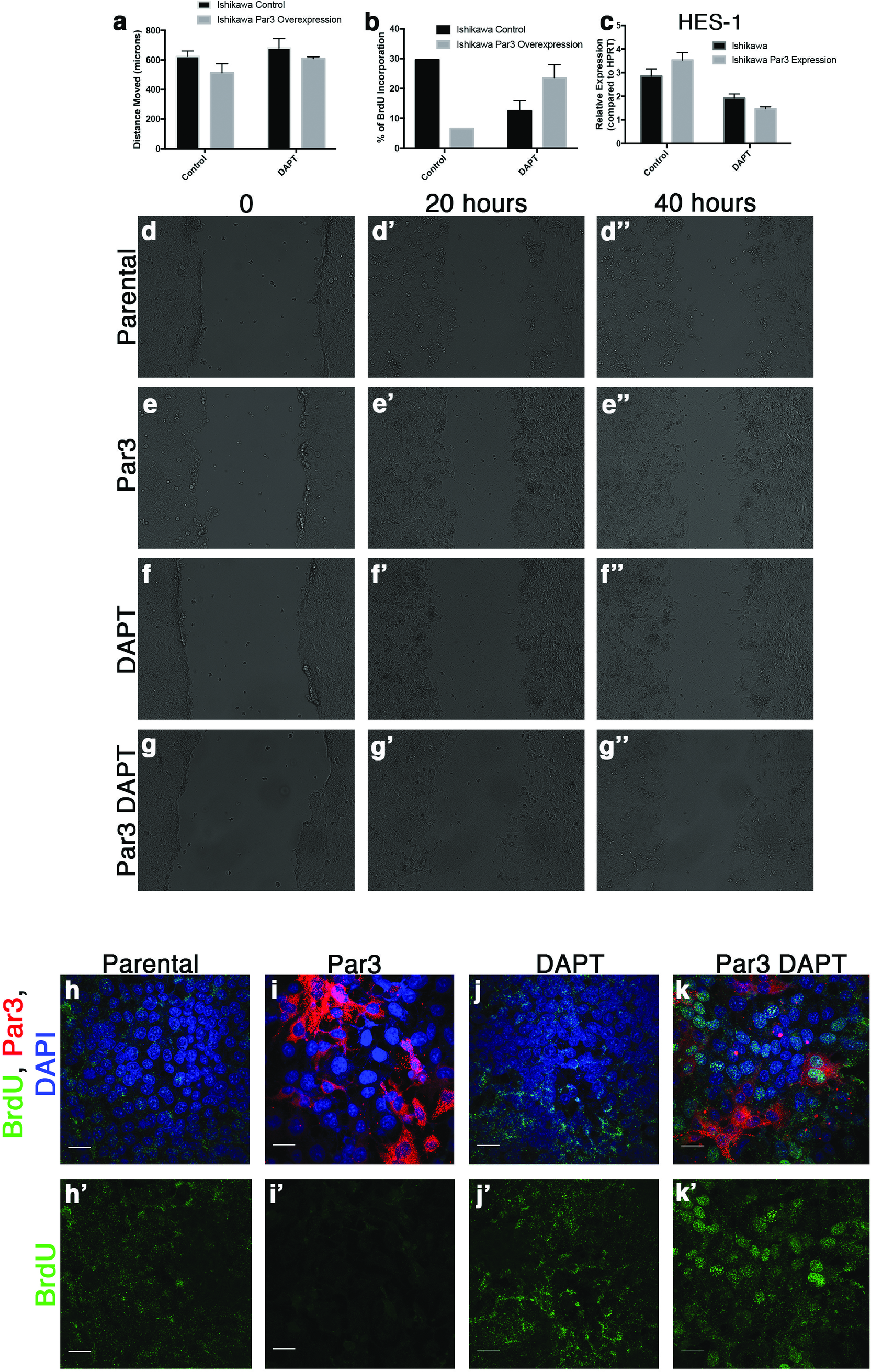

Supplement: S5 Fig — Corresponds to Fig 7. (a) Quantification of cell migration for parental Ishikawa cells, Par3 overexpression Ishikawa cells, and Ishikawa cells treated with DAPT. (b) Quantification of BrdU incorporation in the parental, Par3 overexpression, and DAPT treated Ishikawa cells. (c) qRT-PCR analysis of the Notch target HES-1 in parental, Par3 overexpression and DAPT treated Ishikawa cells. (d-g) Photos showing specific times during the migration assay to examine rate of migration for Ishikawa parental cells (d-d”), Ishikawa cells with Par3 expression (e-e”), Ishikawa parental cells treated with DAPT (f-f”), and Ishikawa Par3 expressing cells treated with DAPT (g-g”). Immunofluorescence analysis of BrdU in parental Ishikawa cells (h, h’), Ishikawa cells overexpressing Par3 (i, i’), parental cells treated with DAPT (j, j’) or Par3 expressing cells treated with DAPT (k, k’). Top panels (h-k) show BrdU (green) with DAPI (blue) staining and panels (h’-k’) show BrdU staining alone. Scale bar, 20 μM. (TIF) [file pone.0189081.s006.tif]
